# Supplementary material for: Microwave emission from superconducting vortices in Mo/Si superlattices
Source: Nat Commun. 2018 Nov 22;9:4927. doi: 10.1038/s41467-018-07256-0 (PMC6250723; doi:10.1038/s41467-018-07256-0)
Supplement: Supplementary file 1 — Supplementary Information [file 41467_2018_7256_MOESM1_ESM.pdf]

Supplementary information for the manuscript "Microwave emission from superconducting vortices in Mo/Si superlattices"

O. V. Dobrovolskiy,[1,2] V. M. Bevz,[2] M. Yu. Mikhailov,[3] O. I. Yuzephovich,[3] V. A. Shklovskij,[4] R. V. Vovk,[4] M. I. Tsindlekht,[5] R. Sachser,[1] and M. Huth[1]

[1]*Physikalisches Institut, Goethe University, Max-von-Laue-Str. 1, 60438 Frankfurt am Main, Germany*
[2]*Physics Department, V. N. Karazin Kharkiv National University, Svobody Sq. 4, 61022 Kharkiv, Ukraine*
[3]*B. I. Verkin Institute for Low Temperature Physics and Engineering of the National Academy of Sciences of Ukraine, Nauky Ave. 47, 61103 Kharkiv, Ukraine*
[4]*Physics Department, V. N. Karazin Kharkiv National University, Svobody Sq. 4, 61077 Kharkiv, Ukraine*
[5]*The Racah Institute of Physics, The Hebrew University of Jerusalem, Givat Ram, 91904 Jerusalem, Israel*

(Dated: October 31, 2018)

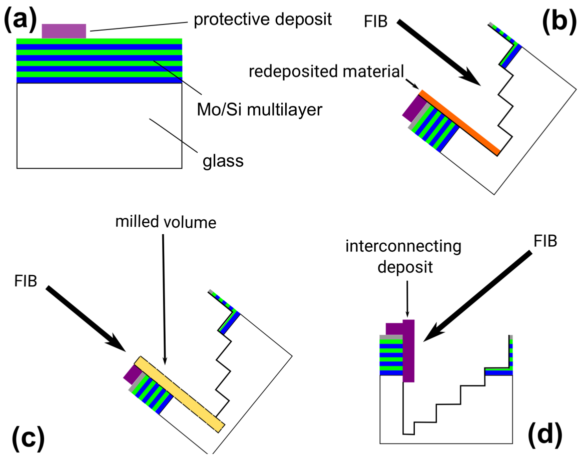

Supplementary Figure 1. Fabrication of contacts. First, a protective Pt-based layer was deposited on top of the Mo/Si multilayer (a) by focused ion beam-induced deposition (FIBID). Next, a stair-like groove was milled by focused ion beam (FIB) under normal beam incidence and then, the sample was tilted by 52° (b). The redeposited layer of Mo-Si-Ga on the side wall of the milled groove was removed by FIB milling ("cleaning cross-section") so that the Mo/Si multilayer structure was uncovered (c). Finally, a conducting Pt-FIBID layer was deposited at the side wall, covering the whole area from the top protective layer down to the substrate (d).

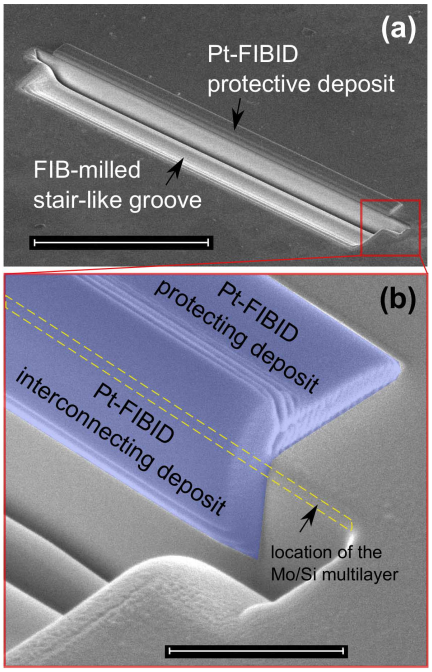

Supplementary Figure 2. Scanning electron microscopy images after contact fabrication steps (c) and (d) in Supplementary Figure 1. The scale bars correspond to 20 μm in panel (a) and 2 μm in panel (b). Panel (b) is a false-color image.

Supplementary Note 1. Vortex lattice configurations at matching fields

The commensurability effect in anisotropic layered superconductors was considered theoretically by Bulaevskij and Clem (BC)[5] on the basis of the discrete Lawrence-Doniach approach and by Ivlev, Kopnin, and Pokrovsky (IKP) in the framework of the continuous Ginzburg-Landau model[6]. BC predicted a sequence of first-order phase transitions between vortex lattices with different matching orders at strong, parallel magnetic fields[5]. The transitions occur at $H_{n,n-1}$ expressed through the characteristic field $H_0 = \Phi_0/\gamma s^2$ at which the overlap of the Josephson cores of vortices is essential. Here, Φ_0 is the magnetic flux quantum, s is the multilayer period, and $\gamma = (M/m)^{1/2} \approx 5.22$ is the anisotropy parameter. It is deduced from the critical field slopes near the superconducting transition temperature T_c, as detailed in the next section. In the expression for γ, M is the effective mass of the Cooper pairs perpendicular to the layer planes while m is the in-plane mass. For our sample $H_0 = 15.8\,\mathrm{T}$ such that a transition between commensurate phases with the vortex lattice period $Z_0 = s$ and $Z_0 = 2s$ should occur in the field $H_{2,1} \approx H_0/3 = 5.27\,\mathrm{T}$. Another transition between the phases with $Z_0 = 2s$ and $Z_0 = 3s$ is expected at $H_{3,2} \approx H_0/8 = 1.975\,\mathrm{T}$. While the field values calculated within the BC model corresponded well to the fields of resistance minima in superlattices with the same $d_\mathrm{Mo} = 22\,\text{Å}$ but a larger $d_\mathrm{Si} = 34\,\text{Å}$ with $\eta_\mathrm{J} \approx 0.7$[4,7], the $R(H^{\parallel b})$ curve of our sample has no minima at the BC matching fields. We attribute this to a larger interlayer coupling in our sample and proceed to a comparison of the data with the continuous IKP model.

IKP showed that when the intrinsic pinning energy exceeds the elastic energy of a vortex lattice shear deformation, the vortices cannot cross the layers[6]. In this case the vortex lattice is always commensurate with the layered structure period s, and the vortex lattice period Z_0 is determined by the initial conditions under which the lattice was formed. Accordingly, $Z_0 = Ns$, where N is an integer, is independent of the external field, while the vortex lattice unit cell area varies with the field only due to vortex displacements along the layers. It was shown that the free energy of the rhombic lattice in the commensurate state as a function of H has two minima corresponding to the different orientations of the unit cell vectors with respect to the layer planes. According to IKP, the conditions of stability, which correspond to the free energy minima, are $2N^2s^2\gamma\sqrt{3}H_N^{(1)} = \Phi_0$ and $2N^2s^2\gamma H_N^{(2)} = \sqrt{3}\Phi_0$, where the stable states of the commensurate lattices correspond to a rhombic lattice with the apex angles $\varphi^{(1)} = 2\pi/3$ and $\varphi^{(2)} = \pi/3$ in the direction of motion. In the instability region there are many metastable states corresponding to different displacements of the vortex rows relative to each other in the neighboring interlayers. These states can be dynamically accessible under the H variation[8].

The IKP matching fields in the data range are $H_{N=1}^{(1)} = 4.2\,\mathrm{T}$ and $H_{N=2}^{(2)} = 3.15\,\mathrm{T}$, in perfect agreement with the field values at which the resistance minima are observed in Fig. 3(c). Accordingly, our analysis of the resistance minima suggests that we deal with a lattice of Abrikosov rather than Josepshon vortices. At the same time, we can not rule out a crossover from Abrikosov to Josephson vortices with further decrease of the temperature, as such a crossover is known in layered systems when the Abrikosov vortex with a suppressed order parameter in its core turns into a Josephson phase vortex once its core completely fits into the insulating layer[9]. Further support in favor of dealing with Abrikosov vortices is provided by the I-V curves allowing for a universal scaling in the flux-flow regime, which would be impossible due to a sudden dissipation reduction at the crossover from Abrikosov to Josephson vortices[9].

Supplementary Note 2. Superconductivity dimensionality crossover in the Mo/Si superlattice

The evolution of the matching minimum in the $R(T)$ curve at $T = 3.6\,\mathrm{K}$ to the zero-resistance state at $T = 1.8\,\mathrm{K}$ can be understood with the aid of the dimensional crossover in the Mo/Si, as inferred from the H-T phase diagram shown in Fig. 5(a). In Fig. 5(a), the temperature dependence of the upper critical field $H_{\mathrm{c}2}(T)$ is plotted for the in-plane and out-of-plane field directions. The $H_{\mathrm{c}2}(T)$ data were deduced from the $R(T)$ curves by the 90% resistance criterion. Near T_c, for both directions $H_{\mathrm{c}2} \propto (1 - T/T_\mathrm{c})$ with slopes of $|\frac{dH_{\mathrm{c}2}^{\parallel c}}{dT}|_{T_\mathrm{c}} = 1.9\,\mathrm{T/K}$ and $|\frac{dH_{\mathrm{c}2}^{\parallel b}}{dT}|_{T_\mathrm{c}} = 10.9\,\mathrm{T/K}$, yielding an anisotropy parameter $\gamma = 5.72$. The out-of-plane upper critical field extrapolated to zero temperature $H_{\mathrm{c}2}^{\parallel c}(0) = 7.4\,\mathrm{T}$ yields $\xi_{ab} = [\Phi_0/2\pi H_{\mathrm{c}2}^{\parallel c}(0)]^{1/2} = 67\,\text{Å}$ and, hence, $\xi_c(0) = \xi_{ab}(0)/\gamma = 12\,\text{Å}$. At lower temperatures $H_{\mathrm{c}2}^{\parallel b} \propto (T_\mathrm{c} - T)^{1/2}$, pointing to a transition at $T^* \approx 3.6\,\mathrm{K}$ from the 3D regime of weak layering with $\xi_c(T) > 70\,\text{Å}$ near T_c to the 2D regime of strong layering at lower temperatures[10].

The increase of the size of the vortex core with increasing temperature $\simeq 2\xi_c(T)$ is illustrated in Fig. 5(b) in comparison with the thickness of the Si layer d_Si and the multilayer period s. A semi-quantitative relation of the vortex core size to the Si layer thickness and the multilayer period is sketched on the top of the spectra in Fig. 2. In particular, at 1.8 K, being the lowest temperature accessible in our experiment, the vortex core $2\xi_c(1.8\,\mathrm{K}) \approx d_\mathrm{Si} = 28\,\text{Å}$ largely fits into the insulating layers, thereby allowing the Mo layers to remain superconducting up to very high fields[11,12]. At 3 K the vortex core $2\xi_c(3\,\mathrm{K}) \approx s \approx 50\,\text{Å}$ becomes comparable with the multilayer period. Even though some part of the vortices penetrates into the Mo layers, there are field ranges where the intrinsic pinning energy E_p is larger than the elastic energy of a vortex lattice shear deforma-

tion E_{el}, which explains the presence of a rather broad resistance minimum in the vicinity of the matching fields. At 3.6 K the vortex cores become appreciably larger than the multilayer period, namely $2\xi_c(3.6\,\mathrm{K}) > 70\,\text{Å}$, such that the intrinsic confinement potential is smoothed out as the vortex core extends over more than one multilayer period. In this case the superlattice is no longer felt by a vortex as a layered structure, but rather the motion of vortices occurs in some effective continuous medium. Accordingly, the matching minimum at 3.15 T becomes shallow at 3.6 K while the minimum at 4.2 T disappears altogether as this field value is too close to $H_{c2}^{\parallel b}(3.6\,\mathrm{K}) = 5.2\,\mathrm{T}$ and it gets smeared by the transition to the normal state.

Supplementary Note 3. Lock-in transition

For the in-plane field geometry in layered superconductors there is a lock-in transition which lifts the restriction for the external magnetic field H to be aligned perfectly parallel to the layer planes in our measurements[1,2]. Namely, Feinberg and Villard have shown that in the general case when H is applied at a finite angle Θ out of planes, there is a finite lock-in angle Θ_{c}, such that when $\Theta < \Theta_{\mathrm{c}}$ the flux lines run strictly parallel to the planes, remaining locked in between the layers[1]. Previous experiments have revealed that in our system $\Theta_{\mathrm{c}} \approx 1^\circ$ at $T = 0.9T_{\mathrm{c}}$, and it increases to $\Theta_{\mathrm{c}} \simeq 2^\circ - 3^\circ$ with decrease of temperature[3]. A further fingerprint for the field tilt angle Θ to be smaller that Θ_{c} is the positions of the resistance minima which do not shift with Θ variation[4]. This has been proven to be the case in our measurements as Θ was varied between -1° and $+1^\circ$. No changes in the emission spectra have been observed at such Θ variation.

Supplementary References

1 D. Feinberg and C. Villard, Phys. Rev. Lett. **65**, 919 (1990).

2 M. Tinkham, *Introduction to Superconductivity* (Mineola, New York, 2004).

3 O. I. Yuzephovich, Y. V. Bomze, M. Y. Mikhailov, I. M. Dmitrenko, and N. Y. Fogel, Low Temperature Physics **26**, 103 (2000).

4 M. Y. Mikhailov, O. I. Yuzephovich, Y. V. Bomze, E. I. Buchstab, and N. Y. Fogel, Low Temp. Phys. **31**, 244 (2005).

5 L. Bulaevskii and J. R. Clem, Phys. Rev. B **44**, 10234 (1991).

6 B. I. Ivlev, N. B. Kopnin, and V. L. Pokrovsky, J. Low Temp. Phys. **80**, 187 (1990).

7 M. Y. Mikhailov, O. I. Yuzephovich, A. S. Pokhila, Y. V. Bomze, N. Y. Fogel, I. M. Dmitrenko, S. A. Yulin, A. S. Sidorenko, O. B. Moldovan, and E. I. Buchstab, Low Temp. Phys. **25**, 635 (1999).

8 L. S. Levitov, Phys. Rev. Lett. **66**, 224 (1991).

9 P. J. W. Moll, L. Balicas, V. Geshkenbein, G. Blatter, J. Karpinski, N. D. Zhigadlo, and B. Batlogg, Nat. Mater. **12**, 134 EP (2012), article.

10 G. Deutscher and O. Entin-Wohlman, Phys. Rev. B **17**, 1249 (1978).

11 P. Berghuis, E. Di Bartolomeo, G. A. Wagner, and J. E. Evetts, Phys. Rev. Lett. **79**, 2332 (1997).

12 K. Iida, J. Hänisch, E. Reich, F. Kurth, R. Hühne, L. Schultz, B. Holzapfel, A. Ichinose, M. Hanawa, I. Tsukada, M. Schulze, S. Aswartham, S. Wurmehl, and B. Büchner, Phys. Rev. B **87**, 104510 (2013).
